# Supplementary material for: Clustered Regularly Interspaced Short Palindromic Repeat Analysis of Clonal Complex 17 Serotype III Group B Streptococcus Strains Causing Neonatal Invasive Diseases
Source: Int J Mol Sci. 2021 Oct 27;22(21):11626. doi: 10.3390/ijms222111626 (PMC8584069; doi:10.3390/ijms222111626)
Supplement: Supplementary file 1 [file ijms-22-11626-s001.zip › ijms-1429390-supplementary.pdf]

Supplementary Material

Clustered Regularly Interspaced Short Palindromic Repeat Analysis of Clonal Complex 17 Serotype III Group B *Streptococcus* Strains Causing Neonatal Invasive Diseases

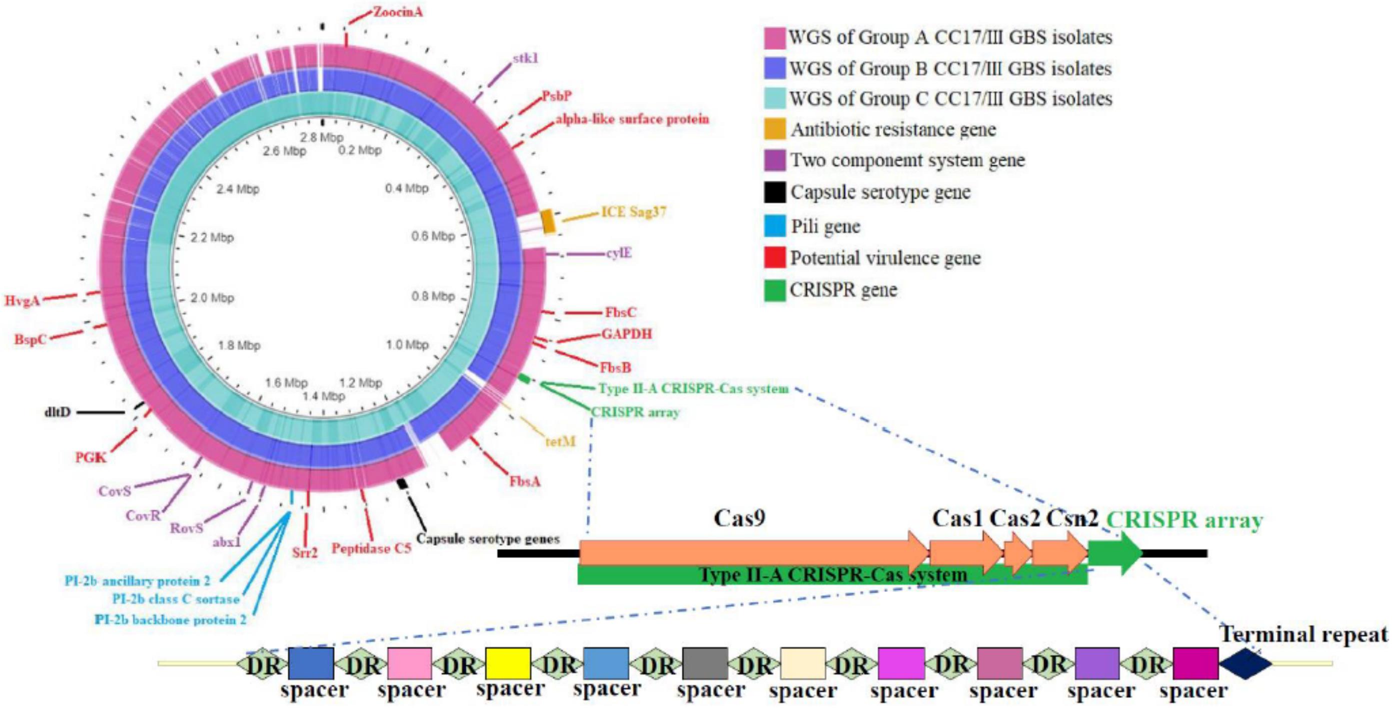

Figure S1. Whole genome sequencing (WGS) analyses of CC17/III GBS isolates from three different CRISPR-RFLP patterns. The potential virulence genes for occurrence of meningitis (red color) are not significantly different between GBS isolates from three subgroups. The only difference is the lack of ICE*Sag37* in group A GBS isolates, which is associated with antimicrobial resistance. DR: direct repeats.

Table S1. Correspondence between spacers and patients' conditions.

	n (%)	Gestational Age (weeks)	Birth Body Weight (g)	Meningitis *n* (%)	Complicated GBS Sepsis *n* (%)	WBC Count (1000/uL)	Hemoglobin Value (g/dL)	C-Reactive Protein (mg/L)	Leukopenia *n* (%)
spacer 56	101 (98.1)	38 ± 3	2950 ± 621	29 (28.7)	33 (32.7)	11,276 ± 7579	11.6 ± 2.4	71.2 ± 84.5	28 (27.7)
spacer 55	97 (94.3)	38 ± 3	2965 ± 595	27 (27.8)	31 (32.0)	11,203 ± 7324	11.6 ± 2.4	68.6 ± 83.9	27 (27.8)
spacer 54	96 (93.2)	38 ± 3	2974 ± 592	27 (28.1)	31 (32.3)	11,102 ± 7295	11.6 ± 2.4	69.2 ± 84.1	27 (28.1)
spacer 53	95 (92.2)	38 ± 3	2968 ± 591	27 (28.4)	31 (32.6)	11,163 ± 7309	11.6 ± 2.4	69.8 ± 84.3	27 (28.4)
spacer 49	93 (90.3)	38 ± 3	2942 ± 591	26 (28.0)	30 (32.3)	11,204 ± 7444	11.6 ± 2.4	69.5 ± 84.3	27 (29.0)
spacer 254	46 (44.7)	38 ± 3	3004 ± 683	12 (26.1)	13 (28.3)	12,067 ± 7337	11.7 ± 2.4	64.8 ± 81.4	7 (15.2)
spacer 243	34 (33.0)	38 ± 3	2880 ± 635	6 (17.6)	9 (26.5)	10,347 ± 7022	11.4 ± 1.9	62.1 ± 71.5	13 (38.2)
spacer S12	6 (5.8)	37 ± 2	2643 ± 500	4 (66.7)	4 (66.7)	4950 ± 3139	10.4 ± 1.5	111.7 ± 62.4	4 (66.7)
spacer S11	5 (4.9)	38 ± 1	2976 ± 370	4 (80.0)	4 (80.0)	5340 ± 3343	10.9 ± 1.2	91.8 ± 43.9	3 (60.0)
spacer S13, S14, S15	5 (4.9)	37 ± 2	2656 ± 557	4 (80.0)	4 (80.0)	5320 ± 3360	10.1 ± 1.5	131.3 ± 44.5	3 (60.0)
spacer S18	3 (2.9)	38 ± 1	2893 ± 474	-	-	14,033 ± 1185	13.0 ± 0.9	100.2 ± 95.6	-
spacer S6	2 (1.9)	40 ± 3	3003 ± 491	1 (50.0)	1 (50.0)	14,950 ± 71	14.2 ± 0.4	36.0 ± 25.2	-
spacer S17	2 (1.9)	35 ± 0	2310 ± 184	2 (100)	2 (100)	650 ± 71	10.3 ± 4.8	70.8 ± 47.1	2 (100)
spacer S1, S10	1 (1.0)	33	1880	-	-	3000	8.1	210.8	1 (100)
spacer 277	1 (1.0)	42	2655	-	-	15,000	14.5	53.9	-
spacer 82, 83, 84, 85, 87, 119, 120, 449, S2, S3	1 (1.0)	38	2920	-	1 (100)	14,100	15.2	101.19	-
spacer 102	81 (78.6)	38 ± 3	2949 ± 634	20 (24.7)	24 (29.6)	10,653 ± 6632	11.5 ± 2.1	62.5 ± 75.5	23 (28.4)
spacer 101	81 (78.6)	38 ± 3	2942 ± 642	19 (23.5)	23 (28.4)	10,578 ± 6832	11.5 ± 2.1	63.4 ± 76.3	24 (29.6)
spacer 984	46 (44.7)	38 ± 3	3004 ± 690	12 (26.1)	13 (28.3)	12,067 ± 7337	11.7 ± 2.4	64.8 ± 81.4	7 (15.2)
spacer 171	31 (30.1)	38 ± 4	2900 ± 757	8 (25.8)	9 (29.0)	13,977 ± 7386	11.9 ± 2.4	52.9 ± 64.1	1 (3.2)
spacer S7	1 (1.0)	40	2900	-	-	20,600	11.1	6.8	1 (100)
spacer 7, 86, 476, S4	1 (1.0)	38	2920	-	1 (100)	14,100	15.2	101.2	-
spacer 724	3 (2.9)	37 ± 2	2847 ± 46	2 (66.7)	2 (66.7)	11,000 ± 8314	10.9 ± 0.2	234.6 ± 197.3	1 (33.3)

spacer S9	2 (1.9)	39 ± 2	3113 ± 350	-	-	12,350 ± 1485	12.2 ± 2.9	3.0 ± 1.4	-
spacer 398	1 (1.0)	38	2920	-	1 (100)	14,100	15.2	101.19	-
spacer S5	1 (1.0)	37	2500	1 (100)	1 (100)	28,00	9.6	112.8	1 (100)
spacer S8	1 (1.0)	40	2900	-	-	20,600	111.1	6.8	-
spacer S19	1 (1.0)	38	3480	-	-	16,000	12.6	0.6	-
